# Electronic structures and dielectric function of (5, 5) CNT-C2H4O system: A first-principles study on the detection capability of CNT for gas sensing applications

**DOI:** 10.55730/1300-0527.3578

**Published:** 2023-07-04

**Authors:** Alvanh Alem G. PIDO, Art Anthony Z. MUNIO

**Affiliations:** 1 Department of Physics, Mindanao State University–Main Campus, 9700 Marawi City, Philippines; 2 Department of Physics, Mindanao State University–Iligan Institute of Technology, A. Bonifacio Avenue, 9200 Iligan City, Philippines; 3 College of Arts and Sciences, Jose Rizal Memorial State University–Tampilisan Campus, 7116 Tamplisan, Zamboanga Del Norte, Philippines

**Keywords:** Carbon nanotubes, metallic armchair, acetaldehyde, density functional theory, Van Hove singularities

## Abstract

Carbon nanotubes (CNTs) are known to have a wide range of applications in various fields of discipline. In this research, the ability of metallic armchair (5, 5) CNT to detect acetaldehyde (C_2_H_4_O) was investigated using first-principles density functional theory (DFT) as implemented in Quantum ESPRESSO with the Generalized Gradient Approximation (GGA). Accordingly, it was found that C_2_H_4_O preserved the metallic behavior of the CNT. However, some bands are found to have overlapped in both the valence and conduction regions of the electronic structures of the resulting system that are mainly caused by the p orbitals of the oxygen and carbon atoms of the compound. These are further confirmed by the projected density of states (pDOS). Moreover, optical transitions are observed in both the real and imaginary parts of the dielectric function caused by the interband transitions between the Van Hove singularities of the electronic structures. In all circumstances, this research has provided more insights into the potential gas sensing applications of metallic CNTs.

## 1. Introduction

The discovery of nanomaterials like carbon nanotubes (CNTs) has unleashed a new era for nanotechnology. CNTs belong to a large group of carbon-based nanomaterials with a radius of up to 50 nm [1–3]. Since its discovery in the previous decades [4], this material has captured the attention of many scholars and received an increasing amount of interest due to its unique and remarkable properties, and wide-ranging applications in materials science and other notable scientific advancements [5]. The intrinsic electronic properties of CNTs are primarily based on their chirality and radius [6–13]. As known, this nanomaterial can be built by rolling up a single layer of graphene, a two-dimensional honeycomb structure of carbon atoms, along the 2D lattice vector given by [14]


(1) $$\vec{R}=n{\vec{R}}_{1}+m{\vec{R}}_{2}$$

where ***n*** and ***m*** are the chiral indices. When ***n* = *m***, the CNT is considered as armchair which theoretically exhibits a metallic behavior. When ***n* – *m =* 3*l ±* 1**, the built CNT exhibits a semiconducting property [15].

With the rising demand of biomedical applications of carbon nanotubes, some investigations were done to see the potential of making efficient CNT-incorporated biosensors and gas sensors [16–20]. These sensors have been widely used to ensure high quality control of numerous diseases and in making sure food safety in the context of food industry. Through the detection of potential hazardous gases and their concentrations, the quality of foods can be controlled. Acetaldehyde is one of the widely occurring compounds in nature that is largely produced in many industries. When in high concentrations, it can cause negative effects on the body [21–24]. In a paper by Mahdavian et al. [25], semiconducting CNT-based on chemical sensors was tested for its sensor response to small concentrations of acetaldehyde and ethyl acetate formation. It was found that the electrical resistance of the semiconducting CNT has changed.

Despite the existing theoretical and computational studies on the sensing ability of the CNTs to acetaldehydes, the potential effect of these gases on the electronic and optical behaviors of CNTs are still unknown. Thus, in this study, the electronic structures and dielectric function of the CNT-C_2_H_4_O system were investigated using first-principles Density Functional Theory (DFT) integrated with the random-phase approximation (RPA) [26]. This study will provide more theoretical insights on the detection capability of CNTs for potential gas sensing applications.

## 2. Computational details

The armchair (5,5) CNT was constructed with 40 carbon (C) atoms. For sidewall adsorption, the compound was placed 2.5 Å from the sidewall of the metallic CNT. The resulting systems were isolated by introducing at least 13 Å vacuum slab perpendicular to the tube axis. This was done to ensure that the interactions of neighboring images are eliminated. For all the calculations, a 500-eV cut-off energy was considered.

**Figure 1:**
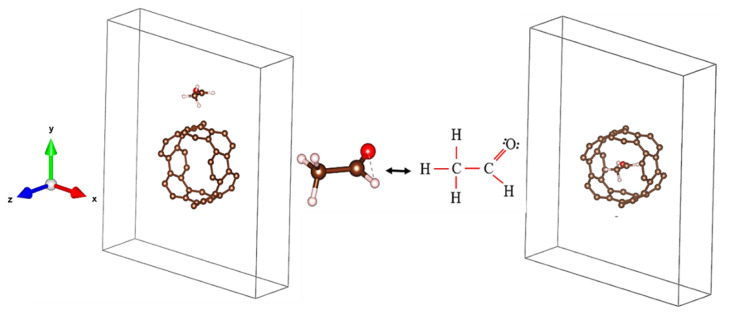
Optimized structures. (a) and (c) are site 1 and site 2, respectively.

In this work, first-principles calculations were employed as implemented in Quantum ESPRESSO [27] with the Generalized Gradient Approximation (GGA) and norm-conserving pseudopotentials from the GBRV library [28]. Along the Γ-Z direction, 60 k points were considered for the electronic band structure calculations while a 1x1x20 k-point sampling was considered for the density of states (DOS). The dielectric function was then calculated using the Random-Phase Approximation (RPA) [26] to investigate the optical transitions of the CNT-C_2_H_4_O system. Figure 1 depicts the optimized systems for site 1 and site 2.

To ensure the stability of interaction, the adsorption energies for both sites were calculated using the formula [13],


(2) $$E_{adsorption}=E_{system\;(site\;1/site\;2)}-E_{CNT}-E_{C_{2}H_{4}O}$$

## 3. Results and discussion

**Figure 2:**
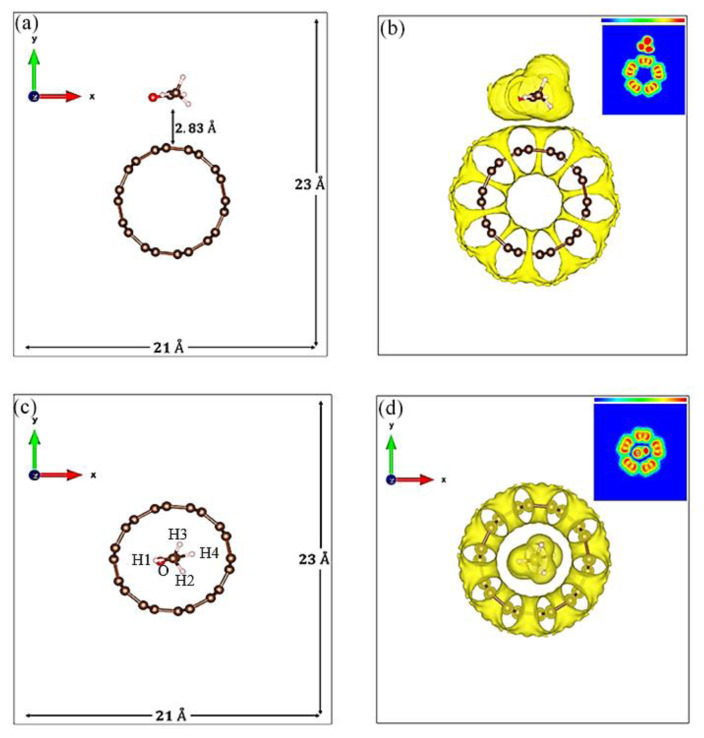
Optimized CNT-C_2_H_4_O systems with their corresponding ELF.

Figure 2 depicts the CNT-C_2_H_4_O systems inside the 21 Å x 23 Å simulation box. The molecular distance between the C_2_H_4_O molecules in the periodic simulation boxes was 2.05 Å. The calculated adsorption energies were −0.22 eV and −0.23 eV for site 1 and site 2 respectively, where the negative sign indicates an exothermic process upon adsorption. Figure 2a shows the optimized structure for C_2_H_4_O sidewall adsorption (site 1). Evidently, the separation distance of the CNT and C_2_H_4_O became 2.83 Å. To understand the binding mechanism of the interaction, the Electron Localization Function (ELF) was calculated [29,30]. Accordingly, the absence of localized electrons between the aldehyde and CNT in Figure 2b indicates a physical binding type of interaction [31,32]. In Figure 2c, the aldehyde was encapsulated in the CNT core (site 2). Here, the interatomic distances of the nearest neighboring atoms between the aldehyde and the CNT are presented in the table. Finally, the corresponding ELF for site 2 shows that the interaction is of physical binding type. This further explains the consistent adsorption energies for both considered sites.

**Figure 3:**
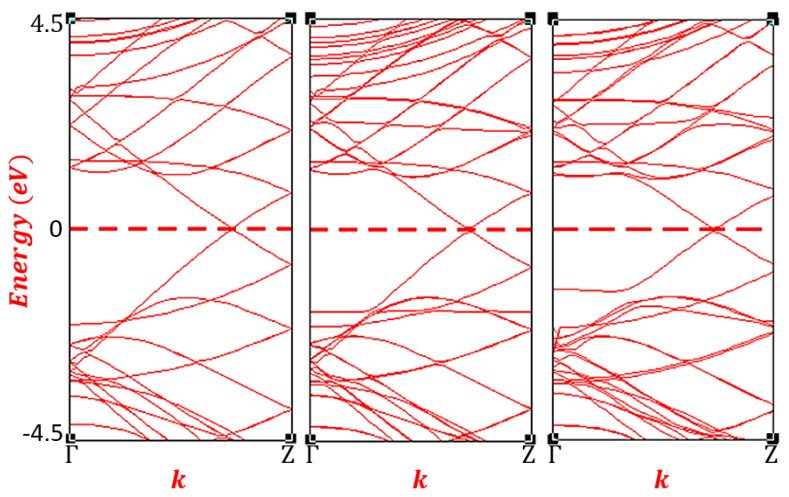
Electronic band structures of (a) pristine and CNT-C_2_H_4_O system (b) site 1 and (c) site 2.

To investigate the charge redistribution and electronic structures of the systems, the band structures and density of states were calculated. Figure 3 shows the electronic band structures of the pristine and CNT-C_2_H_4_O system in the Γ – *Z* direction in the Brillouin zone. In Figure 3a, the crossing of the bands in the Fermi energy level of the pristine (5,5) CNT has confirmed its intrinsic metallic behavior. These crossing of bands have formed Dirac Cones near the Z high symmetry point confirming the findings in the previous literatures [33,34]. In Figure 3b, the band structure of the functionalized CNT was depicted (for site 1). Clearly, no visible changes occur near the Fermi energy level. In fact, the crossing bands were still present, preserving the appearance of Dirac cones. However, at approximately 1.15 eV, some bands are found to have overlapped. This could further be seen above 2.25 eV. This electronic structure is likely due to the interaction of the orbitals of the carbon atoms in the C_2_H_4_O and that of the CNT. Moreover, some charge redistributions are observed in the valence region of the functionalized CNT. At around −2.25 eV, additional bands are visible which is mainly due to the p orbital of the oxygen atom in the C_2_H_4_O. For the C_2_H_4_O encapsulation in the CNT, no evident changes in the band structures are observed except for the small changes in band patterns at the valence region. As shown in Figure 3c, there is a slight increase in energy gap between the highest occupied molecular orbital and the next occupied orbital. This is observed around 2.25 eV.

**Figure 4:**
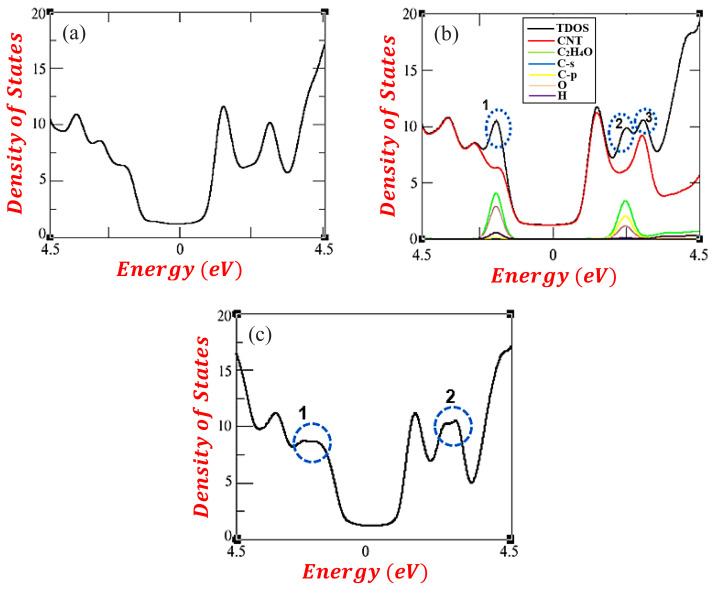
Density of states of (a) pristine and CNT-C_2_H_4_O system (b) site 1 and (c) site. 2.

Figure 4 presents the DOS of the pristine and functionalized CNT. In Figure 4a, the nonzero DOS of the pristine CNT confirms its metallic behavior. Figure 4b, depicts the total and projected density of states of the functionalized CNT (site 1). Consistent with the band structures calculations, no visible changes are present at the Fermi level, retaining the metallicity of the CNT. In the conduction band, some electronic transitions and/or peaks were observed at above 2.25 eV. This is coherent with the overlapping of electronic structures in the previous calculations. As shown in the projected density of states (pDOS), the p orbitals of the carbon atoms in the aldehyde are the main causes of these peaks. Meanwhile, the oxygen atom was found as the main cause of the additional peaks in the valence region. Figure 4c depicts the DOS corresponding to site 2. Accordingly, no visible changes were observed near the Fermi level. Though slight peak changes were seen at both the conduction and valence regions, the height of these peaks are arbitrary, indicating that the electronic structures are similar for C_2_H_4_O encapsulation and sidewall adsorption on (5, 5) CNT.

The dielectric function of the systems was then investigated within the framework of random-phase approximation [35,36]. Mathematically, it is given by


(3) $$ɛ(\omega )={ɛ}_{1}(\omega )+{ɛ}_{2}(\omega )$$

where *ε*_1_(*ω*) and *ε*_2_(*ω*) are the real and imaginary parts of the dielectric function. Here, two directions were considered in the self-consistent calculations. These are the X-X (perpendicular to the tube axis) and Z-Z (parallel to the tube axis) directions in the Brillouin zone.

**Figure 5:**
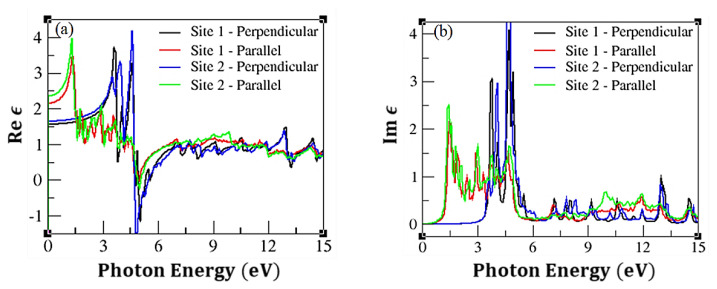
(a) Real and (b) imaginary parts of the dielectric function of CNT-C_2_H_4_O system.

Figure 5a depicts the plot of the photon energy vs. *ε*_2_(*ω*) (or Re *ε*). Clearly, site 1 and site 2 adsorption pose the same optical transition patterns confirming the very small changes in the electronic structures in Figure 3 and Figure 4. Two high peaks are present for the perpendicular direction while one was observed for the parallel direction. These are evident for both site 1 and site 2 adsorption. Despite these differences in peak sizes, more optical transitions were observed below 6 eV for the parallel direction than the perpendicular one. The imaginary part of the dielectric function for both considered sites are shown in Figure 5b. The observed peaks are due to the interband transitions between the Van Hove singularities of the electronic structures [37–42].

On the basis of these findings, it is evident that the interaction of intrinsic metallic CNT with aldehyde compound can significantly modify the electronic and optical configurations of the CNT. In response, the CNT has the capability to detect such gases as seen in electronic and optical transitions of the system. This indicates that CNT has the potential to becoming a good biosensor for organic compounds.

## 4. Conclusion

Investigating the properties of hybrid systems is one of the important ways in determining the potential applications of materials. In this study, the capability of (5,5) CNT to interact with C_2_H_4_O was determined to see what happens to the electronic and optical properties of the resulting system. As shown, charge redistributions and overlapping of bands were observed in the electronic structures of the system. Further, optical transitions are observed due to the interband transitions between the Van Hove singularities of these electronic structures. Based on these findings, it is evident that (5,5) CNT can dramatically detect gases like C_2_H_4_O. In all circumstances this research has provided more insights on the potential biosensing and gas sensing applications of metallic CNTs.

## Figures and Tables

**Table:** Interatomic distances of the nearest neighboring atoms between the aldehyde and the CNT for site 2.

Neighboring atoms	Interatomic distances (Å)
C_CNT_-O	2.89
C_CNT_-H1	2.60
C_CNT_-H2	2.42
C_CNT_-H3	2.44
C_CNT_-H4	2.37
